# Supplementary material for: Controlling long-term SARS-CoV-2 infections can slow viral evolution and reduce the risk of treatment failure
Source: Sci Rep. 2021 Nov 19;11:22630. doi: 10.1038/s41598-021-02148-8 (PMC8604936; doi:10.1038/s41598-021-02148-8)
Supplement: Supplementary file 1 — Supplementary Information. [file 41598_2021_2148_MOESM1_ESM.pdf]

SUPPLEMENTARY INFORMATION FOR

Controlling long-term SARS-CoV-2 infections can slow viral evolution and reduce the risk of treatment failure

Debra Van Egeren, Alexander Novokhodko, Madison Stoddard, Uyen Tran, Bruce Zetter, Michael S. Rogers, Diane Joseph-McCarthy, Arijit Chakravarty*

* Corresponding author: arijit@fractaltx.com (A.C.)

Supplementary Note

Phenotypic changes conferring fitness advantages in SARS-CoV-2 variants

Several variants with phenotypic changes have been characterized. Increased transmissibility (which has been observed *in vivo* with spike protein mutations such as D614G[58]) best explains the rapid spread of B.1.1.7 in multiple countries[5]. Greater lethality has been observed for the B.1.1.7 variant[30] along with faster replication[31]. Increased potential for reinfection is seen for B.1.351[32]. Multiple variants evade antibody therapies: for instance, B.1.526, B.1.429, B.1.427, P.1 and B.1.351 are all resistant to spike-targeting monoclonal antibody bamlanivimab[59]. In other cases, reduced susceptibility to vaccines has been reported. In the extreme case, the variant B.1.351 wholly evades the ChAdOx1 vaccine[37]. *In vitro* work found that sera from people vaccinated with CoronaVac does not neutralize the P.1 variant[60]. This could explain the reported lower efficacy of CoronaVac in clinical trials in Brazil, where P.1 is prevalent, when compared to the United Arab Emirates. *In vivo* data, such as sequencing of breakthrough infections, would strengthen or falsify this hypothesis. Some viral variants have also been associated with longer duration of infection[38], which is particularly concerning in the light of the findings in this report.

This list is not comprehensive, and new data are continually being reported.

Supplementary Table S1. Emergence of currently common SARS-CoV-2 variants

**Variant**	**Site of Emergence**	**Date first reported**	**Local case frequency since emergence as of 4/4/2021**
B.1.351	South Africa	October 2020[61]	1668/2301 (72.5%)[62]
B.1.1.7	UK	September 2020[61]	173624/277811 (62.5%)[62]
P.1	Brazil	January 2021[63]	584/1519 (38.4%)[62]

The B.1.351 variant was first identified in South Africa and quickly became the dominant variant in South Africa and in many other countries. As of April 4th, 2021, the B.1.351 variant accounted for 72.5% of the case frequency in South Africa, and 7 other countries have at least a 72% frequency of B.1.351. This variant is known to increase the transmission rate and moderately impact neutralization by monoclonal antibodies[64].

The B.1.1.7 variant first emerged in the UK in early September 2020. By late December 2020 (week 52 of 2020), B.1.1.7 became the dominant variant in the UK, with a frequency of 51.31%. This variant is known to increase transmissibility and may increase risk of death compared with other variants[5,30].

There are also some shared mutations among these three variants. The P.1. variant and B.1.351 variant share three mutations in the Spike protein (E484K, N501Y, and D614G), while the P.1. variant and B.1.1.7 share the N501Y mutation[64].

Supplementary References

58. Plante, J. A. *et al.* Spike mutation D614G alters SARS-CoV-2 fitness. *Nature* **592**, 116–121 (2021).

59. US Food and Drug Administration. Fact Sheet For Health Care Providers Emergency Use Authorization (EUA) Of Bamlanivimab. 26.

60. de Souza, W. M. *et al.* Levels of SARS-CoV-2 Lineage P.1 Neutralization by Antibodies Elicited after Natural Infection and Vaccination. *SSRN Journal* (2021) doi:10.2139/ssrn.3793486.

61. CDC. Science Brief: Emerging SARS-CoV-2 Variants. *Centers for Disease Control and Prevention* https://www.cdc.gov/coronavirus/2019-ncov/science/science-briefs/scientific-brief-emerging-variants.html (2020).

62. PANGO lineages. https://cov-lineages.org/global_report.html.

63. Another new coronavirus variant found in Japan | The Japan Times. https://www.japantimes.co.jp/news/2021/01/11/national/science-health/new-coronavirus-variant-japan/.

64. CDC. SARS-CoV-2 Variant Classifications and Definitions. *Centers for Disease Control and Prevention* https://www.cdc.gov/coronavirus/2019-ncov/cases-updates/variant-surveillance/variant-info.html (2020).
